# To: Contemporary treatment of children with critical and near-fatal asthma

**DOI:** 10.5935/0103-507X.20160063

**Published:** 2016

**Authors:** José Colleti Junior, Werther Brunow de Carvalho

**Affiliations:** Pediatric Intensive Care Unit, Hospital Santa Catarina - São Paulo (SP), Brazil.; Intensive Therapy/Neonatology, Instituto da Criança, Departamento de Pediatria, Universidade de São Paulo - São Paulo (SP), Brazil.

**To the Editor**

The review article by Shein et al.^(1)^ on the treatment of acute severe asthma in children is timely due to the prevalence of the disease and often variable and inconsistent disease management, which includes adjuvant therapies and depends on the availability of resources, the local practices and the preference of doctors. The risk of death in patients subjected to mechanical ventilation for severe acute asthma was analyzed by Pendergraph et al.,^(2)^ who concluded that the in-hospital mortality rate was 60 to 90 times higher (approximately 10%) for patients who require intubation, with or without admission to an intensive care unit, than for patients who were not intubated. Herein, we discuss two topics related to the review article that can prevent intubation and its complications in severe asthma: the early use of high flow nasal cannula (HFNC) and intravenous magnesium sulfate in the emergency department.

High flow nasal cannula is an emerging therapy for inspiratory effort support that combines an oxygen supply and positive expiratory pressure without the need for complex equipment and with good patient adherence and comfort. In addition to the effect of the positive pressure support, the therapy with HFNC reduces physiological dead space and provides better carbon dioxide clearance. A decrease in the cardiac and respiratory frequency within the first hours after the therapy begins is sufficient to identify patients who respond to this non-invasive support.^(3)^

Although intubation remains the standard for providing invasive respiratory support to critically ill patients (clinically unstable), pulmonary ventilation introduces risks of complications, such as alveolar stress and strain (e.g., volutrauma, barotrauma, biotrauma and endotrauma) and infection. The main goal of non-invasive respiratory support modalities, such as HFNC, is to reduce the need for intubation. Kelly et al.^(4)^ propose that the use of HFNC in the emergency department can result in a lower probability of intubation.

The use of magnesium sulfate can lead to faster resolution of acute severe asthma in children who do not respond adequately to the initial standard treatment,^(5)^ thus avoiding the possibility of intubation and reducing the time of hospital stay. The dose recommended by Shein et al.^(1)^ is 25 to 40 mg/kg intravenously, with infusion for a period between 20 and 30 minutes. However, Irazuzta et al.^(6)^ report that the early use of high doses of magnesium sulfate with continuous infusion (50mg/kg/hour for 4 hours) expedites discharges from the emergency department and prevents hospitalizations in the intensive care unit, with a significant reduction in costs. We used this therapeutic scheme in our unit successfully without any reported adverse effects. Egelund et al.^(7)^ used magnesium sulfate at the dose of 50 - 75mg/kg in a bolus, followed by 40mg/kg/hour for 4 hours, with good results and no significant adverse effects. Ohn et al.^(8)^ suggest that magnesium sulfate should be administered to all children presenting with acute severe asthma. Colleti et al.^(9)^ argue that magnesium sulfate can be a great option in the emergency department to avoid intubation and/or hospitalization, but the optimal dose and the best method of administration still require further study.

We must wait for more research on the use of HFNC and magnesium sulfate in severe acute asthma in various clinical scenarios in pediatrics.

José Colleti Junior

Pediatric Intensive Care Unit, Hospital Santa Catarina - São Paulo (SP), Brazil.

Werther Brunow de Carvalho

Intensive Therapy/Neonatology, Instituto da Criança, Departamento de Pediatria, Universidade de São Paulo - São Paulo (SP), Brazil.
